# A surveillance system to assess the need for updating systematic reviews

**DOI:** 10.1186/2046-4053-2-104

**Published:** 2013-11-14

**Authors:** Nadera Ahmadzai, Sydne J Newberry, Margaret A Maglione, Alexander Tsertsvadze, Mohammed T Ansari, Susanne Hempel, Aneesa Motala, Sophia Tsouros, Jennifer J Schneider Chafen, Roberta Shanman, David Moher, Paul G Shekelle

**Affiliations:** 1 Knowledge Synthesis Group, Ottawa Hospital Research Institute, Clinical Epidemiology Program, Center for Practice-Changing Research, 501 Smyth Road, Ottawa, ON K1H 8L6, Canada; 2 Southern California Evidence-based Practice Center (SCEPC), The RAND Corporation, 1776 Main Street, PO Box 2138, Santa Monica, CA 90401, USA; 3 Stanford University, Stanford University 117 Encina Commons, Stanford, CA 94305-6019, USA; 4 Veterans Affairs Greater Los Angeles Healthcare System, 11301 Wilshire Boulevard, Los Angeles, CA 90073, USA

**Keywords:** Systematic review, Updating, Surveillance

## Abstract

**Background:**

Systematic reviews (SRs) can become outdated as new evidence emerges over time. Organizations that produce SRs need a surveillance method to determine when reviews are likely to require updating. This report describes the development and initial results of a surveillance system to assess SRs produced by the Agency for Healthcare Research and Quality (AHRQ) Evidence-based Practice Center (EPC) Program.

**Methods:**

Twenty-four SRs were assessed using existing methods that incorporate limited literature searches, expert opinion, and quantitative methods for the presence of signals triggering the need for updating. The system was designed to begin surveillance six months after the release of the original review, and thenceforth every six months for any review not classified as being a high priority for updating. The outcome of each round of surveillance was a classification of the SR as being low, medium or high priority for updating.

**Results:**

Twenty-four SRs underwent surveillance at least once, and ten underwent surveillance a second time during the 18 months of the program. Two SRs were classified as high, five as medium, and 17 as low priority for updating. The time lapse between the searches conducted for the original reports and the updated searches (search time lapse - STL) ranged from 11 months to 62 months: The STL for the high priority reports were 29 months and 54 months; those for medium priority reports ranged from 19 to 62 months; and those for low priority reports ranged from 11 to 33 months. Neither the STL nor the number of new relevant articles was perfectly associated with a signal for updating. Challenges of implementing the surveillance system included determining what constituted the actual conclusions of an SR that required assessing; and sometimes poor response rates of experts.

**Conclusion:**

In this system of regular surveillance of 24 systematic reviews on a variety of clinical interventions produced by a leading organization, about 70% of reviews were determined to have a low priority for updating. Evidence suggests that the time period for surveillance is yearly rather than the six months used in this project.

## Background

Systematic reviews (SRs) on the effectiveness and safety of various health interventions are the basis for clinical practice guidelines, public and corporate policy, and clinical and consumer decision-making. These SRs provide systematically searched, collected, evaluated, and synthesized scientific evidence to objectively compare the effectiveness, benefits, and safety of different health interventions. The production of SRs is based on standardized, structured, and explicit methodological guidance. The SRs endeavor to focus on patient-relevant outcomes (for example, mortality, pain, quality of life, functional status, myocardial infarction) in addition to relevant intermediate surrogate outcome measures (for example, cholesterol levels, serum glucose levels, red blood cell count) [1].

Systematic reviews may be conducted by independent groups of researchers or by researchers associated with large organizations such as the Cochrane Collaboration; the United States Agency for Healthcare Research and Quality (AHRQ), which administers a group of Evidence-based Practice Centers (EPC) throughout North America; and the National Institute for Health and Clinical Excellence (NICE) in the UK [2]. A primary responsibility of these organizations is the conduct of systematic reviews, the results of which are often posted on their websites.

The inevitable - and rapid - accumulation of new research findings has raised concern among these organizations about how best to identify which reviews may be out of date and whether to sponsor an update or simply remove the outdated review from their websites. To date, organizations and initiatives (for example, Cochrane Collaboration, Drug Effectiveness Review Project (DERP)) have relied on time-based (for example, annual, biennial) periodic updating policies that have proven to be problematic in terms of feasibility and efficiency [3-5]. However several lines of evidence demonstrate that reviews become obsolete at different rates, suggesting that a system of regular surveillance might be a more effective way of identifying potentially out-of-date reviews. In 2006, the DERP implemented a strategy for assessing the need for updating systematic reviews of comparative effectiveness and safety of drug interventions evaluated in controlled clinical trials [6]. The DERP’s stakeholders need to make coverage decisions for new drugs, and therefore the appearance of a new drug is a strong signal for an update. However, not all SR users (for example guideline developers) might consider a new drug within an established class (such as a new statin or angiotensin receptor antagonist) as an indication of the need for an update. Furthermore, SRs may deal with non-pharmacologic interventions (for example, diagnostic screening) and include observational studies.

AHRQ supported a pilot study comparing different methods to assess signals for the need to update SRs and another study to assess an initial set of SRs that were considered Comparative Effectiveness Reviews (CERs) for the need to update. CERs are systematic reviews that aim to compare the benefit and harms of a range of options rather than only answering a narrow question on safety and effectiveness of a single therapy [2]. Based on these pilot studies, AHRQ supported the development of a surveillance system for regularly monitoring AHRQ’s portfolio of SRs. This article presents the results of the surveillance system covering June 2011 to November 2012.

## Methods

### The surveillance system - summary overview

**Figure 1:**
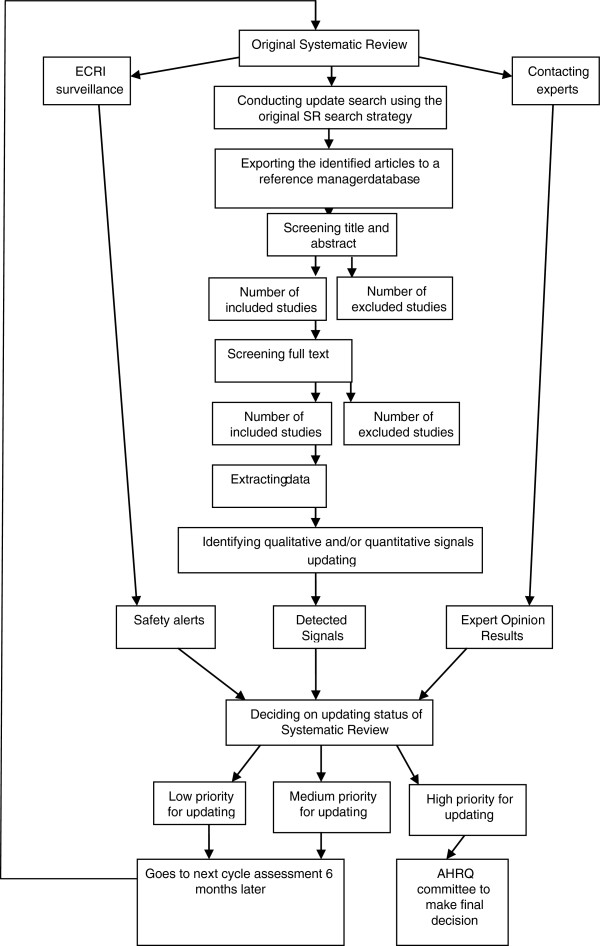
**The process of surveillance assessment for a systematic review (SR).** Figure 1 portrays the overall process of surveillance assessment for an SR that mainly includes: 1) literature search, 2) contacting experts, and 3) obtaining safety alerts from various sources sent by ECRI (one of the AHRQ evidence-based centers). The number of hits identified by literature search would be transferred to Reference Manager database and then will be screened by: 1) title and abstract, and 2) full text. The data was extracted from the number of studies that were deemed eligible for inclusion. Next, the extracted data was assessed for identifying qualitative and quantitative signals. Then, the findings from literature, expert opinion, and safety alerts were collated and assessed for updating priority status (high, medium or low). If an SR was deemed as ‘high’ priority for assessment, it was referred to AHRQ for updating. If an SR was deemed as ‘medium’ or ‘high’ priority for updating, it was re-assessed six months after the completion of the first assessment.

Two EPCs (RAND, University of Ottawa) participated in the development of the surveillance system; a third EPC (ECRI) assisted in obtaining safety alerts). The RAND and Ottawa EPCs had independently developed methods to assess SRs for the need to update [7,8]; a formal comparison of the two showed they produced similar results [9]. In developing and implementing the surveillance system, we operationalized a proposal made in our earlier CER surveillance report for what such a system would look like (see Figure 1). This article describes the surveillance assessment of 24 consecutive SRs conducted for the AHRQ Effective Health Care’s Comparative Effectiveness Review program [10-33].

The surveillance system was designed to conduct an assessment of a SR six months after its release and every six months thereafter until the assessment identified signals sufficient to classify it as ‘high priority’ for an update. Briefly, six months after release of a SR, we conduct abbreviated literature searches, using the strategy employed in the original SR, but limited to five general medicine journals and approximately five specialty journals specific to the topic of the SR and, with a few exceptions. Newly identified evidence relevant to the key questions and the original conclusions was abstracted, and pre-specified criteria were used to detect the presence of qualitative and/or quantitative signals for updating (EPC SRs are organized around a set of key questions, each of which might have multiple parts, resulting in the need for multiple conclusions) [7]. The method also incorporates expert opinion regarding the validity or currency of conclusions reached in the SR and government safety alerts relevant to the SR [8]. Based on a combination of the weight of the evidence, signals, and expert opinion, a determination was made regarding the need to update each conclusion for each key question, with the expectation that a change in conclusion may yield a change in clinical practice. That is, each key question (KQ)-specific conclusion within a SR was categorized as up-to-date, possibly out-of-date, probably out-of-date, or out-of-date [8]. Finally, based on: 1) the proportion of key questions whose conclusions were determined to require updating or the urgency to update a particular set of conclusions, and 2) the extent of outdatedness, a global assessment of priority status was assigned to updating the full report (high, medium, or low), and the results of the process were summarized in a brief report. SRs assigned a low or medium priority for updating were re-assessed six months later. Reports assigned a high priority for updating were not re-assessed. The decision to update or withdraw the report is made by AHRQ, who consider the availability of resources and other factors when making a final decision. Detailed methods of the surveillance process are presented as the following:

### Abbreviated search, study selection, and data extraction

The ascertainment of updating signals relied on qualitative and/or quantitative criteria developed originally for the Ottawa method [7] and expert opinion as used in the RAND method [8]. For each SR, we conducted an abbreviated update search as described in previous publications [7,9,34]. We employed the strategies used in the original published SRs but limited the sources searched to five general medical journals (*Annals of Internal Medicine*, *BMJ*, *JAMA*, *The Lancet*, and *New England Journal of Medicine*) and approximately five topic-specific specialty journals (usually the journals that contributed the most evidence to the original report; (if a particular specialty journal was not catalogued in PubMed, we would search the more relevant database as well). These searches were conducted for a time period starting six months prior to the last date covered by the searches for the original SR (to minimize the number of relevant studies missed due to delayed publication) up to the present. We also assessed the eligibility of studies referenced by content experts (further detail on the experts in the following sections). After removing duplicates from identified records, one reviewer used the inclusion/exclusion criteria specified in the original SR to screen titles and abstracts and then full texts of potentially relevant records. For each included new study, one reviewer extracted relevant data on study characteristics (for example, design, sample size, follow-up duration), demographic factors for study participants (for example, age, sex, condition), treatment (for example, type, frequency, dose), outcome characteristics, and results into an evidence table.

### Ascertainment of updating signals

To identify signals/triggers for updating, we applied qualitative and/or quantitative criteria [7] to the abstracted evidence for each conclusion in the original SR. For each conclusion, we first documented the absence of new evidence (that is, no new evidence or new evidence showing the same or similar conclusion as the original SR) or the presence of new evidence meeting the pre-defined criteria of signal(s) indicating a need for updating (Table 1).

**Table 1:** Criteria for determining that a conclusion is out-of-date

**Ottawa’s label**	**Ottawa method**
	**Qualitative criteria for potentially invalidating signals**
A1	Opposing findings: a pivotal^*^ trial or systematic review (or guidelines) including at least one new trial that characterized the treatment in terms opposite to those used earlier
A2	Substantial harm: a pivotal trial or systematic review (or guidelines) whose results called into question the use of the treatment based on evidence of harm or that did not proscribe use entirely but did potentially affect clinical decision-making
A3	A superior new treatment: a pivotal trial or systematic review (or guidelines) whose results identified another treatment as significantly superior to the one evaluated in the original review, based on efficacy or harm
	**Qualitative criteria for signals of major changes**
A4	Important changes in effectiveness short of ‘opposing findings’
A5	Clinically important expansion of treatment
A6	Clinically important caveat
A7	Opposing findings from discordant meta-analysis or non-pivotal trial
	**Quantitative criteria signals of changes in evidence**
B1	A change in statistical significance (from nonsignificant to significant)
B2	A change in relative effect size of at least 50 percent
**RAND’s label**	**RAND method indications for the need for an update**
1	Original conclusion is still valid and this portion of the original report does not need updating. This conclusion was reached if we found no new evidence or only confirmatory evidence and all responding experts assessed the CER conclusion as still valid, we classified the CER conclusion as still valid
2	Original conclusion is possibly out-of-date and this portion of the original report may need updating. This conclusion was reached if we found some new evidence that might change the CER conclusion, and/or a minority of responding experts assessed the CER conclusion as having new evidence that might change the conclusion, then we classified the CER conclusion as possibly out-of-date
3	Original conclusion is probably out-of-date and this portion of the original report may need updating. This conclusion was reached if we found substantial new evidence that might change the CER conclusion, and/or a majority of responding experts assessed the CER conclusion as having new evidence that might change the conclusion, then we classified the CER conclusion as probably out-of-date
4	Original conclusion is out-of-date. This conclusion was reached if we found new evidence that rendered the CER conclusion out-of-date or no longer applicable; we classified the CER conclusion as out-of-date. Recognizing that our literature searches were limited, we reserved this category only for situations where a limited search would produce *prima facie* evidence that a conclusion was out-of-date, such as the withdrawal of a drug or surgical device from the market, a black box warning from FDA, and so on

*Abbreviation*: *CER* comparative effectiveness review, *FDA* Food and Drug Administration.

*a pivotal trial is defined as trial that is published in one of the top five general medical journals or a trial whose sample size is at least triple that of the largest trial in the original systematic review.

Legend: Table 1 presents the criteria used to determine if a conclusion is out of date within an SR (here CER). Criteria A1 to B2 come from the Ottawa method and criteria 1 to 4 are based on the RAND method.

We then assessed whether new evidence provided or contributed to a qualitative or quantitative signal. One example of a qualitative signal might include finding a newly published pivotal trial with results opposite to that of the original SR with respect to an efficacy outcome (for example, effective versus ineffective or *vice versa*) or a harm (for example, a newly identified risk of harm that outweighs the previously observed benefits). The original definition of a pivotal trial was one published in one of the top five general medical journals or a trial whose sample size was at least triple that of the largest trial in the original SR [7]. For this application we made some adaptations to account for key questions for which observational studies were the study design of choice; namely we did not require new large cohort studies to have at least three times the number of participants as existing large cohorts. Other examples of qualitative signals included a superior new treatment (for example, a new treatment significantly more effective than one assessed in the SR); or a new population subgroup (that is, the treatment assessed in the SR has subsequently been tested on a new population). In contrast, new evidence generates a quantitative signal if its incorporation into a SR’s original meta-analysis changes a statistically non-significant pooled estimate into a statistically significant one or *vice versa*[7].

### Clinical content experts

We identified and contacted two sets of clinical experts: a) those who had worked on the SR in question (for example, the project lead, clinical lead, members of the technical expert panel, and peer reviewers) and b) other clinical experts in the clinical content area who had not worked on the SR in question (for example, local or external subject matter experts). For each SR, we created a matrix that included each of the original key questions and a summary of each conclusion in the original report. Respondents were asked to provide their opinions on whether or not each conclusion was still valid. They were also asked to provide reference citations for any new studies they were aware of that might invalidate or otherwise alter the conclusion(s) as well as studies that were pertinent to the topic but might not address a particular conclusion directly (for example, studies of newer treatments that may have rendered the original treatments out-of-date). The responding experts were offered a small honorarium; reminders were sent to experts who did not initially respond.

### Safety alerts

We examined safety and adverse event alerts relevant to each SR. This information was collected from MedWatch, the US Food and Drug Administration’s Safety Information and Adverse Event reporting system; the UK’s Medicines and Health Care Products Regulatory Agency (MHRA); and Health Canada.

### Determination of updating status for SRs

The information on updating signals, expert opinion, and safety alerts was collated, summarized, and tabulated. Taking into consideration the totality of evidence, we used a set of decision rules/guidance originally used in the pilot studies [8,9] to characterize any given KQ-related conclusion(s) as up-to-date, possibly out-of-date, probably out-of-date, or out-of- date. Based on the totality of these characterizations, each SR was assigned to high, medium, or low updating priority groups. The decision to assign a high priority was not based strictly on the proportion of conclusions determined to be probably or definitely out-of-date, but rather, was a global judgment informed by a set of guidelines; for example, one out-of-date conclusion that could result in harm or inferior treatment could give rise to a high priority for updating. The criteria for determining updating status are provided in Additional file 1.

For each of the SRs that underwent surveillance, we summarized our findings in a brief report. These reports are now posted on the AHRQ website along with the original SRs to which they refer.

### Assessment of the findings across SRs

To gain a sense of how long it takes for SRs to go out-of-date, we assessed the proportion of the SRs that went through the surveillance process at least once that received a high or medium priority for updating as a function of the length of time since their publication and from the date of their latest searches.

## Results

### Sampling of SRs for assessment

Between June 2011 and November 2012, we assessed 24 SRs at least once. When we implemented the surveillance system, a backlog of SRs had accumulated and needed to be assessed. In addition, there was a 3- to 17- month lag between the completion of the original or update search and the release of the reports. Thus, there was a time span of 11 to 62 months from the completion of the original or update searches and the surveillance search (Table 2).

**Table 2:** Characteristics of 24 comparative effectiveness reviews (CERs) and their associated updating surveillance assessments

**CER title; first author last name (publication date)**	**Latest search date for CER (across databases)**	**Number of included studies in CER (total or per KQ)**	**Period covered by surveillance assessment search**	**Number of new studies judged as relevant for inclusion in CER**
**(Journal publication, if available)**	**Time between search and report release**		**Time between original searches and update search**	
Comparative Effectiveness of Therapies for Clinically Localized Prostate Cancer; Wilt (February 2008) [22]	September 2007	436	January 2007 to March 2012	21
(Systematic review: association Between Hospital and Surgeon Radical Prostatectomy Volume and Patient Outcomes: A Systematic Review) [35]	5 months		54 months	
Comparative Effectiveness of Medications to Reduce Risk of Primary Breast Cancer in Women; Nelson (September 2009) [11]	January 2009	13 (KQ1,KQ3)	January 2008 to July 2011	3
(Systematic review: comparative effectiveness of medications to reduce risk for primary breast cancer) [36]	8 months	70 (KQ2,KQ3)	31 months	
		24 (KQ4)		
		16 (KQ5)		
Comparative Effectiveness of Core Needle Biopsy and Open Surgical Biopsy for Diagnosis of Breast Lesions; Bruening (December 2009) [10]	September 2009	107 (KQ1-2)	January 2008 to September 2011	19
(Systematic review: comparative effectiveness of core-needle and open surgical biopsy to diagnose breast lesions) [37]	3 months	NA (KQ3)	24 months	
Effectiveness of Recombinant Human Growth Hormone (rhGH) in the Treatment of Patients with Cystic Fibrosis; Phung (October 2010) [12]	April 2010	26(KQ1-2, KQ4, KQ6-7); 50 (KQ3); 3(KQ5)	January 2010 to August 2011	16
(Systematic review: recombinant human growth hormone in the treatment of patients with cystic fibrosis) [38]	6 months		16 months	
Therapies for Children with Autism Spectrum Disorders; Warren (April 2011) [13]	May 2010	159	January 2009 to October 2011	15
(Systematic review: a systematic review of early intensive intervention for autism spectrum disorders) [39]	11 months		17 months	
Comparative Effectiveness of Traumatic Brain Injury and Depression;	June 2010	115	January 2010 to October 2011	29
Guillamondegui (April 2011) [15]	10 months		16 months	
Pain Management Interventions for Hip Fracture; Abou-Setta (May 2011) [14]	December 2010	98	January 2008 to November 2011	1
(Systematic review: comparative effectiveness of pain management interventions for hip fracture: a systematic review) [40]	5 months		11 months	
Diagnosis and Treatment of Obstructive Sleep Apnea in Adults; Balk (August 2011) [27]	September 2010	44 (KQ1); 1 (KQ2); 2 (KQ3); 11 (KQ4); 173 (KQ5); 6 (KQ6); 18 (KQ7)	January 2010 to April 2012	35
(Systematic review: auto-titrating versus fixed continuous positive airway pressure for the treatment of obstructive sleep apnea: a systematic review with meta-analyses) [41]	11 months		19 months	
Nonpharmacologic Interventions for Treatment-Resistant Depression in Adults; Gaynes (September 2011) [30]	18 November 2010	64	January 2010 to March 2012	9
	10 months		16 months	
Attention Deficit Hyperactivity Disorder (ADHD): Effectiveness of Treatment in At-Risk Preschoolers; Long-term Effectiveness in All Ages; and Variability in Prevalence, Diagnosis, and Treatment; Charach (October 2011) [33]	31 May 2010	53 (KQ1); 76 (KQ2); NR (KQ3)	January 2010 to June 2012	17
	17 months		25 months	
Effectiveness of Early Diagnosis, Prevention, and Treatment of *Clostridium difficile* Infection; Butler (December 2011) [29]	June 2010 and for KQ3 May 2011	13 (KQ1); 36 (KQ2); 13 (KQ3); 40 (KQ4)	January 2010 to June 2012	7
(Systematic review: comparative effectiveness of *Clostridium difficile* treatments: a systematic review) [42]	7 to 18 months		24 months for KQ 1,2, 4	
			13 months for KQ3	
Noncyclic Chronic Pelvic Pain Therapies for Women: Comparative Effectiveness; Andrews (January 2012) [28]	May 2011	23 (KQ1); 7 (KQ2); 0 (KQ3); 17 (KQ4); 0 (KQ5)	May 2011 to July 2012	2
(Systematic review: systematic review of therapies for noncyclic chronic pelvic pain in women) [43]	8 months		14 months	
Chronic Kidney Disease Stages 1 to 3: Screening, Monitoring, and Treatment;	January 2011	110	January 2011 to August 2012	20
Fink (January 2012) [31]	12 months		19 months	
(Systematic review: screening for, monitoring, and treatment of chronic kidney disease stages 1 to 3: a systematic review for the US Preventive Services Task Force and for an American College of Physicians Clinical Practice Guideline) [44]				
First and second generation antipsychotics for children and young adults; Seida (February 2012) [32]	February 2011	81	January 2011 to August 2012	19
(Systematic review: antipsychotics for children and young adults: a comparative effectiveness review) [45]	12 months		18 months	
Comparative Effectiveness of Management Strategies for Renal Artery Stenosis: 2007 Update; Balk (November 2007) [26]	23 April 2007	8	October 2006 to June 2012	7
	7 months		62 months	
Comparative Effectiveness of Radiofrequency Catheter Ablation for Atrial Fibrillation; IP (July 2009) [18]	December 2008	120	June 2008 to September 2011	33
(Systematic review: comparative effectiveness of radiofrequency catheter ablation for atrial fibrillation) [46]	7 months		35 months	
Comparative Effectiveness of Lipid-Modifying Agents; Sharma (September 2009) [17]	May 2009	101	November 2008 to October 2011	20
(Systematic review: comparative effectiveness and harms of combination therapy and monotherapy for dyslipidemia) [47]	4 months		29 months	
Comparative Effectiveness of Angiotensin Converting Enzyme Inhibitors or Angiotensin II Receptor Blockers Added to Standard Medical Therapy for Treating Stable Ischemic Heart Disease; Coleman (October 2009) [20]	February 2009	60	August 2008 to November 2011	12
(Systematic review: comparative effectiveness of angiotensin-converting enzyme inhibitors or angiotensin II-receptor blockers for ischemic heart disease) [48]	8 months		33 months	
Comparative Effectiveness of In-Hospital Use of Recombinant Factor VIIa for Off-Label Indications versus Usual Care; Yank (May 2010) [16]	August 2009	74	February 2009 to January 2012	15
(Systematic review: benefits and harms of in-hospital use of recombinant factor VIIa for off-label indications) [49]	9 months		29 months	
Comparative effectiveness and safety of radiotherapy treatments for head and neck cancer; Samson (May 2010) [19]	September 2009	108	March 2009 to August 2011	7
	8 months		23 months	15
Comparative Effectiveness of Nonoperative and Operative Treatments for Rotator Cuff Tears; Sedia (July 2010) [21]	September 2009	137	March 2009 to January 2012	
(Systematic review: nonoperative and operative treatments for rotator cuff tears) [50]	10 months		28 months	
Comparative Effectiveness of Terbutaline Pump for the Prevention of Preterm Birth; Gaudet (September 2011) [23]	April, 2011	14	October 2010 to March 2012	0
(Systematic review: effectiveness of Terbutaline Pump for the Prevention of Preterm Birth. A Systematic Review and Meta-Analysis) [51]	5 months		11 months	
Self-Measured Blood Pressure Monitoring: Comparative Effectiveness; Uhlig (January 2012) [24]	19 July 2011	48 (KQ1-2); 1( KQ5)	January 2011 to August 2012	1
	6 months	0 (KQ 3–4)	13 months	
Hematopoietic Stem-Cell Transplantation in the Pediatric Population; Ratko (February 2012) [25]	17 August 2011	251	February 2011 to September 2012	0
	6 months		13 months	

*Abbreviations*: *CER* Comparative Effectiveness Review, *KQ* key question, *NR* not reported.

Legend: Columns 1 to 3 present characteristics of CERs:

Column 1: title of the CER, first author’s last name, and title of the journal publication for the same CER.

Column 2: the date that last search was executed for the original CER (for instance, 17 August 2011 refers to the last literature search date carried out for CER), and the time lag between the last search date and the release date of the original CER on the AHRQ website; for example, six months shows the CER was published/released on the AHRQ website six months after the last search date was executed for the CER.

Column 3: total number of studies included in the original CER; for example 251 means a total of 251 studied had met the inclusion criteria of the original CER and were reported in the original CER.

Column 4 presents: 1) time period that the surveillance assessment search has covered (for instance, February 2011 to September 2012 means the search was limited to the period between these dates), and 2) time lag between the original CER search date and the surveillance assessment search date (for example, 13 months means the surveillance literature search was carried out 13 months after the last search date of the original CER).

Column 5 reports the number of studies include in the surveillance assessment; for instance, (15) shows that a total of 15 studies deem eligible and were reported in the surveillance assessment.

### Characteristics of SRs

The SRs varied widely in the kinds of interventions they tested and their target populations. Interventions included pharmaceuticals [11-14,16,17,20-23,28,31,33], surgical procedures [10,18,21,22,25,26,28], radiotherapy [19,22], non-pharmacological procedures [24,28,30], diagnostic and preventive interventions [27,29], and a complementary and alternative medicine intervention [14]. The populations of interest included patients with cancer, tumors, and anomalies on screening [10,11,19,22,25], heart disease [18,20], cystic fibrosis [12], autism [13], trauma [15,16], cuff tears [21], lipid therapy[17], hypertension [24], renal diseases [26,31], attention deficit hyperactivity disorder [33], psychiatric and behavioral conditions [32], depression [30], infection [29], pelvic pain [28], sleep apnea [27], and preterm labor [23].

The characteristics of the 24 SRs and the corresponding surveillance assessments are presented in Table 2. Briefly, the number of key questions (the questions that frame AHRQ SRs) across the 24 SRs ranged from three [10,17,26,33] to seven [12,13,27], although each key question could comprise any number of subquestions. The total number of conclusions per report that required assessment, a reflection of the number of subquestions, ranged from 7 to 86 (the median number was 23). The median number of included studies in the original SRs was 104 (IQR: 71 to 124). The median number of newly identified studies deemed relevant for inclusion in the SRs was 15 (range: 0 to 35).

The number of experts initially contacted across the 24 SRs ranged from 4 [17] to 17 [30]. The response rates ranged from 20% (2/10) to 100% (6/6) with a median of 35%.

Of the 24 SRs, nine [37%] were considered up-to-date as defined by agreement that all conclusions for all key questions were still up-to-date [12,15,21,23-25,28,30,31]. For the remaining 15 (63%) SRs [10,11,13,14,16-20,22,26,27],[29,32,33], at least one conclusion was rated as ‘probably/possibly out-of-date’ or ‘out-of-date.’ For four (17%) SRs [10,19,20,22], all conclusions within at least one key question were rated as ‘probably/possibly out-of-date’ or ‘out-of-date’ (see Table 3).

**Table 3:** Currency of individual conclusions within each key questions of the of 24 comparative effectiveness reviews (CERs) and their priority status for updating (high, medium, and low) based on the updating surveillance assessments

**CER title author name (publication date)**	**Number of conclusions within the key questions in**								**Updating priority for the CER**
	**CER by updating status**								**(low, medium, and high)**
	**KQ#**	**# Conclusions Up-to-date**	**KQ#**	**# Conclusions Possibly out-of-date**	**KQ#**	**# Conclusions Probably out-of-date**	**KQ#**	**# Conclusions Out-of-date**	
Comparative Effectiveness of Therapies for Clinically Localized Prostate Cancer; Wilt (February 2008) [22]	1	11/15	1	2/15			1	2/15	High
							2	1/1	
	3	3/3							
	4	1/3					4	2/3	
Comparative Effectiveness of Medications to Reduce Risk of Primary Breast Cancer in Women; Nelson (September 2009) [11]	1	4/6			1	2/6			Medium
	2	6/7			2	1/7			
	3	4/5			3	1/5			
	4- 5	9/9							
Comparative Effectiveness of Core Needle Biopsy and Open Surgical Biopsy for Diagnosis of Breast Lesions; Bruening (December, 2009) [10]	1	10/16	1a	4/16			1	2/16	Medium
	2	3/4	2	1/4					
	3	1/2	3	1/2					
Effectiveness of Recombinant Human Growth Hormone (rhGH) in the Treatment of Patients with Cystic Fibrosis; Phung (October 2010) [12]	1-7	40/40							Low
Therapies for Children with Autism Spectrum Disorders; Warren (April 2011) [13]	1	10/14	1	4/14					Low
	2	2/3	2	1/3					
	3-7	6/6							
Comparative Effectiveness of Traumatic Brain Injury and Depression; Guillamondegui (April 2011) [15]	1-6	15/15							Low
Pain Management Interventions for Hip Fracture; Abou-Setta (May 2011) [14]	1	7/8	1	1/8					Low
Diagnosis and Treatment of Obstructive Sleep Apnea in Adults; Balk (August 2011) [27]	1	3/4	1	1/4					Medium
	2	1/1							
	3	1/1							
	4	1/1							
	5	14/15	5	1/15					
	6	1/1							
	7	1/1							
Nonpharmacologic Interventions for Treatment-Resistant Depression in Adults; Gaynes (September 2011) [30]	1a	1/1							Low
	1b	1/1							
	2	1/1							
	3	1/1							
	4	1/1							
	5	1/1							
	6	1/1							
Attention Deficit Hyperactivity Disorder (ADHD): Effectiveness of Treatment in At-Risk Preschoolers; Long-term Effectiveness in all Ages; and Variability in Prevalence, Diagnosis, and Treatment; Charach (October 2011) [33]	1	2/3	1	1/3					Low
	2	5/6	2	1/6					
	3	10/12			3	2/12			
Effectiveness of Early Diagnosis, Prevention, and Treatment of *Clostridium difficile* Infection; Butler (December 2011) [29]	1	2/3	1	1/3					Low
	2	6/8	2	2/8					
	3	6/7	3	1/7					
	4	4/5	4	1/5					
Noncyclic Chronic Pelvic Pain Therapies for Women: Comparative Effectiveness; Andrews (January 2012) [28]	1	5/5							Low
	2	6/6							
	3	1/1							
	4	6/6							
	5	1/1							
	all	2/2							
Chronic Kidney Disease Stages 1 to 3: Screening, Monitoring, and Treatment; Fink (January 2012) [31]	1-6	25/25							Low
First and Second Generation Antipsychotics for Children and Young Adults; Seida (February 2012) [32]	1	4/7	1	2/7			1	1/7	Low
	2	3/3							
	3	4/4							
	4	1/1							
Comparative Effectiveness of Management Strategies for Renal Artery Stenosis: 2007 Update; Balk (November 2007) [26]	1	7/15	1	8/15					Medium
	2	2/3	2	1/3					
	3	4/4							
Comparative Effectiveness of Radiofrequency Catheter Ablation for Atrial Fibrillation; IP (July 2009) [18]	1	4/4							Low
	2	3/5	2	2/5					
	3	3/4	3	1/4					
	4	6/6							
Comparative Effectiveness of Lipid-Modifying Agents; Sharma (September 2009) [17]	1	3/13					1	10/13	High
	2	34/48	2	14/48					
	3	9/25	3	16/25					
Comparative Effectiveness of Angiotensin Converting Enzyme Inhibitors or Angiotensin II Receptor Blockers Added to Standard Medical Therapy for Treating Stable Ischemic Heart Disease; Coleman (October 2009) [20]	1	6/7	1	1/7					Low
	2-6	28/28							
					7	4/4			
Comparative Effectiveness of In-Hospital Use of Recombinant Factor VIIa for Off-Label Indications versus Usual Care; Yank (May 2010) [16]	2	2/3	2	1/3					Low
	3a	7/9	3a	2/9					
	3b	3/4	3b	1/4					
	4a	1/2	4a	1/2					
	4b-c	9/9							
Comparative Effectiveness and Safety of Radiotherapy Treatments for Head and Neck Cancer; Samson (May 2010) [19]	1	2/3	1	1/3					Medium
	2	1/2	2	1/2					
			3	1/1					
	4	3/3							
Comparative Effectiveness of Nonoperative and Operative Treatments for Rotator Cuff Tears; Sedia (July 2010) [21]	1- 6	18/18							Low
Comparative Effectiveness of Terbutaline Pump for the Prevention of Preterm Birth; Gaudet (September 2011) [23]	1-6	37/37							Low
Self-Measured Blood Pressure Monitoring: Comparative Effectiveness; Uhlig (January 2012) [24]	1	8/8							Low
	2	4/4							
	3	4/4							
	4	2/2							
	5	2/2							
Hematopoietic Stem-Cell Transplantation in the Pediatric Population; Ratko (February 2012) [25]	1	3/3							Low
	2	3/3							
	3	5/5							
	4	5/5							
	5	5/5							
	6	5/5							

*Abbreviations*: *CER* Comparative Effectiveness Review, *KQ* key question

Legend: Column 1 reports the title and first author of the CERs assessed.

Column 2 reports the currency of each conclusion within each key questions of each CER. For example; CER [22] had a total of four key questions. Key question 1 had a total of 15 conclusions, of which 11 were assessed to be up-to-date, 2 possibly out-of-date and 2 out-of-date based on the surveillance assessment. Column 3 demonstrates the final conclusion (updating priority status) of surveillance assessment for individual CER, for example, if a CER is assessed as high priority for updating, the column shows ‘high’. The symbol # refers to “number” inside the table.

Most SRs were assigned a low priority for updating: two out of 24 SRs (8%) [17,22] were assigned a ‘high’ priority for updating; five out of 24 (21%) [10,11,19,26,27] were assigned a medium priority, and the remaining 17 (71%) were assigned a low priority [12-16,18,20,21,23-25,28-33] (see Table 3).

Ten SR topics underwent a second surveillance assessment. For those SRs, we contacted only those experts who had responded in the first round. Across these ten SRs, 39 experts were contacted, and 27 responded, with response rates ranging from 40% to 100%. Median response rate was 71%, double the 35% median response rates across all topics on the first round. Across these ten SRs that underwent a second surveillance assessment at about six months from the end of the prior assessment, there were 265 conclusions contained within 53 key questions. Of these, eight conclusions changed between the first and second surveillance: seven conclusions changed from ‘up-to-date’ to ‘possibly out-of-date’, and one conclusion changed from ‘possibly out-of-date’ to ‘probably out-of-date’. One of the ten SRs changed priority for updating from ‘low’ to ‘medium’.

### Factors associated with priority decisions

**Figure 2:**
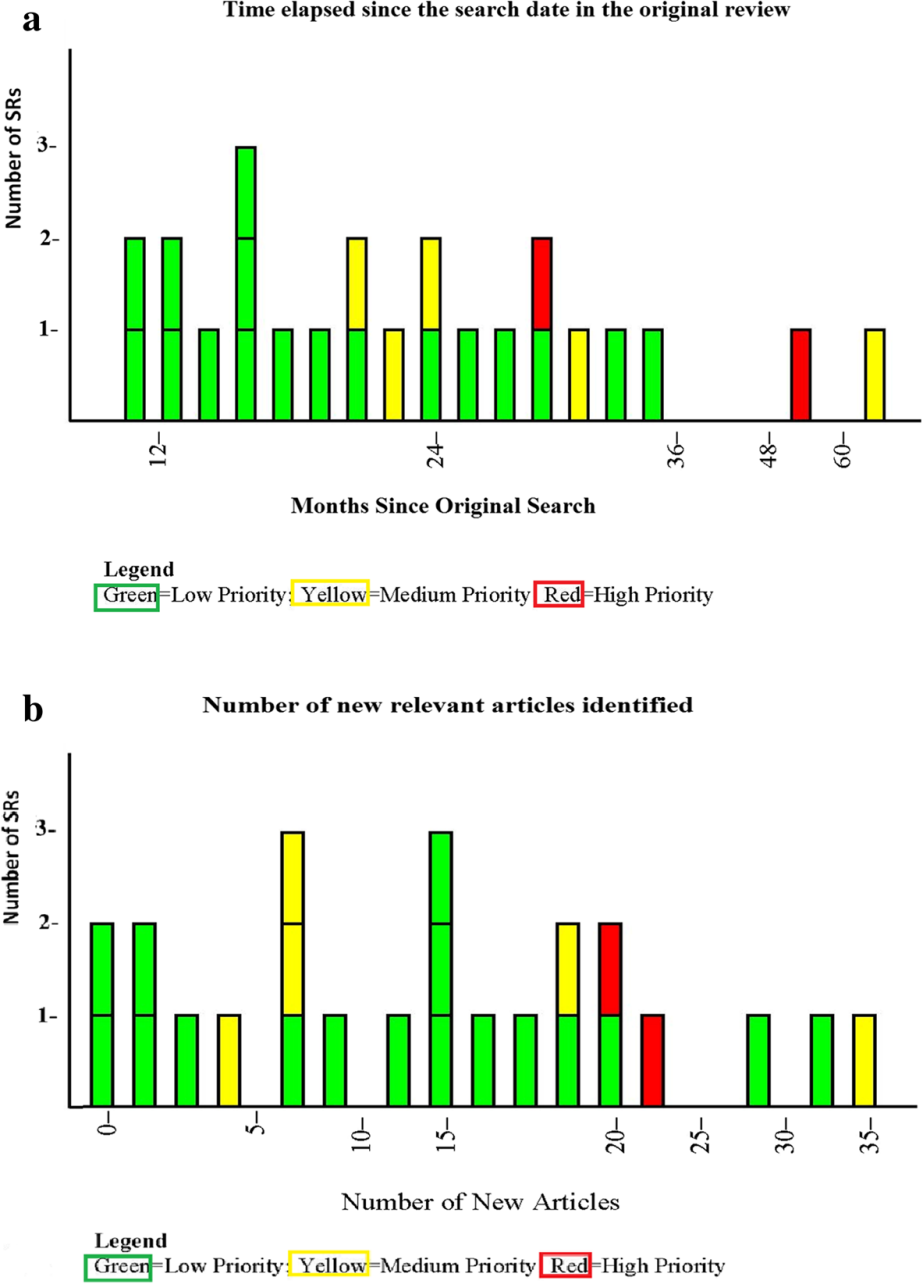
**The process of surveillance assessment for a Systematic Review. (a)** Time elapsed since the search date in the original review. Green color: low priority for updating; Yellow color: medium priority for updating; red color: high priority for updating. **(b)** Number of new relevant articles identified. Green color: low priority for updating; Yellow color: medium priority for updating; red color: high priority for updating.

We assessed whether the length of time that had elapsed between the search conducted for the original report and the update surveillance search (search time lapse, STL) was associated with priority status for updating. Seven SRs were released prior to January 2010 [10,11,17,18,20,22,26] (that is, more than 18 months before the start of the Surveillance Program); of these seven, two were the SRs judged as being ‘high’ priority for updating, three were judged as being ‘medium’ priority, and two were judged as being ‘low’ priority for updating. Of the remaining 17 SRs, released after January 2010, only two were judged as being ‘medium’ priority for updating and the rest were low priority. All SRs released within the year prior to the start of the Surveillance Program (between June 2010 and June 2011) were judged as being ‘low’ priority. Figure 2a and b present the updating priority decisions for the 24 SRs by the time elapsed since the search date in the original review (2a) and the number of new relevant articles identified during the surveillance process (2b). While more SRs were classified as medium or high priority for updating as both the STL and the number of new relevant articles increased, there was substantial overlap, and no threshold existed for either time or number of articles that could accurately predict classification of SRs into different categories.

### The possible role of safety alerts

We identified applicable safety alerts for 9 of the 24 SRs assessed. FDA provided alerts for all nine of those SRs [12,13,15,17,20,27,28,31],[33]; MHRA and Health Canada were the sources of alerts for only one SR [33]. None of the agents, devices, or procedures evaluated in the 24 SRs for which we performed the surveillance assessments had an FDA black box warning (the strongest FDA warning, indicating a significant risk of serious or even life-threatening adverse effect) issued during our assessment period. In only one case was the updating priority of a SR influenced by a safety alert [27].

## Discussion

Our results indicate that a small proportion of AHRQ-supported SRs may need updating within one to two years of the date of their last search. Of the 24 SRs assessed between June 2011 and November 2012, 17 (71%) were classified as having low priority for updating, and five SRs (21%) had medium priority for updating. Only two SRs (8%) were deemed to have high priority. Greater elapsed time from the end date of the original search and a larger number of new relevant studies were both associated with a higher priority for updating, but no thresholds were identified that could perfectly classify SRs into priority categories. This finding suggests that expert opinion will be a necessary component of an efficient system of searching for signals for updating.

Several of the SRs were classified as low priority for updating despite having a large number of newly identified potentially relevant studies. One explanation for this finding is that, in general, many of these new studies had small sample sizes or few primary outcomes and the results were consistent with those of the original SRs, thus not justifying updating those existing SRs. Conversely, the presence of a single new study with many outcome events can be a sufficient signal of the need for a high priority update, such as the publication of the Prostate Cancer Intervention Versus Observation Trial (PIVOT) [52] and the SR on therapies for clinically localized prostate cancer [22].

A recent study that examined factors that predicted 69 decisions on whether to update 41 reviews of drug effectiveness found that the number of relevant new studies was a significant predictor of a decision to update a review (OR 1.06 for each new trial) [6]. This study, conducted for the Drug Effectiveness Review Project (DERP), was designed to examine the surveillance process implemented in 2006 to replace what had been a policy of mandatory annual updates. The DERP process is qualitatively similar to our surveillance method, in that it uses limited literature searches, information from FDA and Health Canada, and expert input. The study also found that identification of a new drug significantly increased the likelihood of an update (OR = 5.71) and that reviews of psychiatric drugs were always recommended for an update. The authors did not report whether there were thresholds of articles or time that perfectly predicted decisions for updating. A major difference between that study and ours, aside from our broader focus on all types of clinical interventions, is that the decision to update a DERP report rests with a panel of participants comprising physicians and representatives of the state Medicaid agencies and the Canadian Agency for Drug Technology and Health, for whom the appearance of a new drug requires them to make policy decision.

### Lessons learned

The implementation of the surveillance assessment program to determine the currency of published AHRQ SRs has presented a number of challenges. These challenges included differences across reports in the ways conclusions were presented, the responsiveness of report staff and experts, and delays in the release of the original reports themselves combined with differences in the length of time between release and surveillance.

#### Inconsistency in presentation of conclusions

Not all SRs presented their KQs and the corresponding conclusions in the executive summary in a similar manner (that is, the degree of detail, format, or level of summarization may have varied). For example, in some SRs, conclusions were, by necessity, stratified by subpopulation, intervention, outcome, or other study characteristics, resulting in multiple conclusions for a single question. In some SRs, the executive summaries failed to present sufficient detail to enable reviewers to extract at least one specific, clearly formulated conclusion for each key question; therefore, the reviewers had to probe the entire text of the SR report. Conversely, some executive summaries simply reproduced the results from the report text without drawing any conclusions, leaving the experts to whom we sent the information to draw their own conclusions. Some conclusions were not readily amenable to updating, for example, conclusions regarding the prevalence of certain risk factors in specific populations.

#### Responsiveness of report staff and experts

Conducting the surveillance on schedule required that the project leads for the original reports and the experts they recommended we contact respond in a timely manner. However, project leads and experts varied widely in their responsiveness to our requests. In addition, response rates were low in the first surveillance. However, it is unclear what this low response means, since the sample is not intended to be a random sample of some larger population. In the second round of surveillance, the response rate improved considerably, suggesting that over time, the surveillance process will become more efficient.

#### Delays in release of some reports

In several cases, surveillance was delayed because a report was not released on schedule. The primary impact of such delays was on our staff’s ability to plan their work schedules, as they would have reserved time for these reports and would need to find other surveillance work or work on our own evidence reviews when a report expected for surveillance failed to materialize.

### Limitations

One limitation of the surveillance system is that it requires subjective global judgments. The assessment of currency and validity of conclusions for each key question in a SR was based on the totality of information compiled through multiple sources such as the qualitative/quantitative signals, expert opinion, and safety alerts. Although we used operational and standardized definitions throughout the process to promote consistency in the assessments, the overall judgment must necessarily be subjective in characterizing individual conclusions. However, since neither the STL nor the number of new relevant studies can classify SRs perfectly as low, medium, or high priority status for updating, this subjective human assessment is going to be needed in an efficient surveillance system. Future work should seek to make these judgments as reliable as possible across raters. The strength of evidence should be investigated in future work.

A second limitation is that we present data for only 34 surveillance assessments on 24 SRs. However, only two published evaluations have included more assessments than ours. A study by Shojania and colleagues assessed 100 systematic reviews to determine how quickly they go out-of-date, but this study limited its sample to meta-analyses that produced a summary estimate of outcome, and then further limited the analysis to only one outcome per study [7]. The DERP study reported the results of surveillance on 41 of their reports [6], but these reports assessed only drugs, and the decisions about updating were made by stakeholders for whom the approval of a new drug was highly relevant to policy decision-making. Our study, by contrast, assesses a broad array of health care interventions, and considered changes in evidence that might lead to changes in practice as the criterion for a signal for updating.

In sum, we found that only a small proportion of AHRQ-sponsored systematic reviews triggered signals for updating within one or two years of the date of their last search, and that neither the elapsed time since the original search nor the number of new articles could perfectly predict which SRs may be in need of updating. Our experience also provided some evidence into what might be the optimal time for a first assessment and subsequent surveillance assessments. Among the 24 SRs released within the first 18 months of surveillance, only two were classified as high priority, five were classified as medium, and the rest were classified as ‘low’ priority for updating (and a number of these reports had been released up to four years prior to the start of the surveillance). Furthermore, there were few changes in conclusions about updating in a second round of surveillance timed to start six months after the completion of the first round. These results suggest to us that a one-year time period between the release of a report and its first and subsequent surveillance assessments may be more efficient than the six-month time frame chosen for this application.

## Conclusion

By undertaking periodic evaluation of 24 topically diverse SRs commissioned by a leading organization, we established the feasibility of a surveillance system to monitor SR currency for a wide range of therapeutic interventions. About 70% of reviews were determined to have a low priority for updating. Evidence suggests that the optimal interval for surveillance is yearly.

For future research, we recommend: 1) modifying and testing the current surveillance methodology to encompass reviews of diagnostic and prognostic methods; 2) validating the surveillance methods against the gold standard of actual review updates in a blinded fashion; and 3) identifying predictors of a review being out-of-date; for example, review quality or the strength of evidence for each individual conclusion; and 4) assessment of the relationship between the quality or strength of evidence and signal detection.

## Abbreviations

AHRQ: Agency for Healthcare Research and Quality; CER: comparative effectiveness reviews; DERP: Drug Effectiveness Review Project; ECRI: Emergency Care Research Institute; EPC: Evidence-based Practice Center; FDA: Food and Drug Administration; IQR: Interquartile range; KQ: Key question; MHRA: Medicines and Health Care Products Regulatory Agency; NICE: National Institute for Health and Clinical Excellence; OR: Odds ratio; SR: Systematic review; STL: Search time lapse.

## Competing interests

The authors declare that they have no competing interests.

## Authors’ contributions

NA, SJN, DM, and PS: 1) have made substantial contributions: a) to conception and design, b) acquisition of data, and c) analysis and interpretation of data; 2) have been involved in: a) drafting the manuscript, and b) revising it critically for important intellectual content; and 3) have given final approval of the version to be published. MM contributed in 1a-c, 2b, and 3. AT carried out 1b-c, 2a-b, and 3. MTA was involved in 1a, 1c, 2b, and 3. SH participated in 1b-c, 2b, and 3. AM participated in 1b and 2 to 3. ST participated in 1b-c, 2b, and 3. JJSC contributed in 1b, 2b, and 3. RH contributed in 1b, 2b, and 3.All authors read and approved the final manuscript.

## Supplementary Material

Additional file 1 Decision rules for determining updating status of a CER conclusion. Click here for file

## Disclaimer

This project was funded under contract number HHSA-290-2007-10062I (RAND Southern California Evidence-based Practice Center) and HHSA-290-2007-10059I (University of Ottawa Evidence-based Practice Center) from the Agency for Healthcare Research and Quality, US Department of Health and Human Services. The authors of this report are responsible for its content. Statements in the report should not be construed as endorsement by the Agency for Healthcare Research and Quality or the US Department of Health and Human Services.
