# Supplementary material for: A surveillance system to assess the need for updating systematic reviews
Source: Syst Rev. 2013 Nov 14;2:104. doi: 10.1186/2046-4053-2-104 (PMC3874670; doi:10.1186/2046-4053-2-104)
Supplement: Additional file 1 — Decision rules for determining updating status of a CER conclusion. [file 2046-4053-2-104-S1.pdf]

Additional File 1:

Decision rules for determining updating status of a CER conclusion*

- "If we found no new evidence or only confirmatory evidence and all responding experts assessed the CER conclusion as still valid, we classified the CER conclusion as still up-to-date.

- If we found some new evidence that might change the CER conclusion, and /or a minority of responding experts assessed the CER conclusion as having new evidence that might change the conclusion, then we classified the CER conclusion as possibly out of date.

- If we found substantial new evidence that might change the CER conclusion, and/or a majority of responding experts assessed the CER conclusion as having new evidence that might change the conclusion, then we classified the CER conclusion as probably out of date.

- If we found new evidence that rendered the CER conclusion out of date or no longer applicable, we classified the CER conclusion as out of date. Recognizing that our literature searches were limited, we reserved this category only for situations where a limited search would produce prima facie evidence that a conclusion was out of date, such as the withdrawal of a drug or surgical device from the market, a black box warning from FDA, etc."

Consideration for assigning the updating priority to a CER

- How many conclusions of the CER are up-to-date, possibly out of date, or certainly out of date?

- How out of date are conclusions (e.g., consideration of magnitude/direction of changes in estimates, potential changes in practice or therapy preference, safety issue including withdrawn from the market drugs/black box warning, availability of a new treatment)

* See Shekelle 2009 AHRQ report (8)
